# Supplementary material for: Analysis of PPARγ Signaling Activity in Psoriasis
Source: Int J Mol Sci. 2021 Aug 10;22(16):8603. doi: 10.3390/ijms22168603 (PMC8395241; doi:10.3390/ijms22168603)
Supplement: Supplementary file 1 [file ijms-22-08603-s001.zip › Supplemental materials_Analysis of PPARg signaling activity in psoriasis/Pathway models/Models images and html files/Differentiation of psoriatic T cells/7175.html]

SMAD2


# Protein SMAD2

|  |  |
| --- | --- |
| URN | urn:agi-llid:4087 |
| Total Entities | 0 |
| Connectivity | 3420 |
| Name | SMAD2 |
| Description | SMAD family member 2 |
| Notes | The protein encoded by this gene belongs to the SMAD, a family of proteins similar to the gene products of the Drosophila gene 'mothers against decapentaplegic' (Mad) and the C. elegans gene Sma. SMAD proteins are signal transducers and transcriptional modulators that mediate multiple signaling pathways. This protein mediates the signal of the transforming growth factor (TGF)-beta, and thus regulates multiple cellular processes, such as cell proliferation, apoptosis, and differentiation. This protein is recruited to the TGF-beta receptors through its interaction with the SMAD anchor for receptor activation (SARA) protein. In response to TGF-beta signal, this protein is phosphorylated by the TGF-beta receptors. The phosphorylation induces the dissociation of this protein with SARA and the association with the family member SMAD4. The association with SMAD4 is important for the translocation of this protein into the nucleus, where it binds to target promoters and forms a transcription repressor complex with other cofactors. This protein can also be phosphorylated by activin type 1 receptor kinase, and mediates the signal from the activin. Alternatively spliced transcript variants have been observed for this gene. [provided by RefSeq, May 2012] |
| Primary Cell Localization | Nucleus |
| Class | Transcription factor |
| ObjectType | Protein |

---

|  |  |
| --- | --- |
| Pathway | Osteoarthritis |
|  | Immunoglobulin Class-Switch Recombination Activation |
|  | anti-Inflammatory Function of Macrophage M2 Lineage |
|  | Vascular Smooth Muscle Cell/Pericyte Differentiation and Proliferation |
|  | PRL Expression Regulation by BMP4 and TGFB1 |
|  | Activin, Inhibin and Follistatin Action on Pituitary Gland |
|  | Cell Cycle Overiew |
|  | Cardiomyocyte Dysfunction |
|  | Dilated Cardiomyopathy |
|  | Cardiomyocyte Hypertrophy |
|  | Hypertrophic Cardiomyopathy |
|  | BMP/TGF-beta Signaling Impairment in Pulmonary Hypertension |
|  | Proteins Involved in Myocardial Ischemia |
|  | TGF-beta Signaling in Skin Aging |
|  | Tubular Cell Dysfunction in Progressive Diabetic Nephropathy |
|  | Endothelial Cell Dysfunction in Progressive Diabetic Nephropathy |
|  | Mesangial Cell Dysfunction in Diabetic Nephropathy |
|  | Prolactinoma Overiew |
|  | Metastatic Colorectal Cancer |
|  | Peutz-Jeghers Syndrome |
|  | Chronic Myeloid Leukemia |
|  | Diffuse Large-B-Cell Lymphoma |
|  | Hodgkin Lymphoma |
|  | Vascularization in Hepatocellular Carcinoma |
|  | NOTCH Signaling in Hepatocellular Carcinoma |
|  | Hepatocellular Carcinoma |
|  | Growth Factor Signaling in Hepatocellular Carcinoma |
|  | Proteins Involved in Pancreatic Cancer |
|  | Pancreatic Ductal Carcinoma |
|  | Pancreatic Neuroendocrine Tumors |
|  | STK11 Signaling in Pancreatic Neoplasms |
|  | TGFBR Signaling in Pancreatic Neoplasms |
|  | Osteoblast Function Decline in Gout |
|  | TGFB2 Signaling Impairement in Osteoarthritis |
|  | Synovial Fibroblast Proliferation in Rheumatoid Arthritis |
|  | Skin Fibrosis |
|  | Hereditary Hemorrhagic Telangiectasia |
|  | Myostatin in Skeletal Muscles in Muscular Dystrophies |
|  | Myostatin-IGF1 Crosstalk in Skeletal Muscles in Muscular Dystrophies |
|  | Bone Loss in Osteoporosis |
|  | Hyperparathyroidism, Secondary Effect |
|  | Glioma Stem Cell Program Activation |
|  | Medulloblastoma |
|  | Proteins Involved in Breast Cancer Related to NOTCH Signaling Pathway |
|  | Proteins Involved in Breast Cancer Related to IGF1R/Akt Signaling Pathway |
|  | Breast Cancer |
|  | NOTCH1 Signaling in Breast Cancer |
|  | Proteins Involved in Female Infertility |
|  | Ovarian Cancer |
|  | INHA Gene Mutations in Granulosa Cell Proliferation |
|  | FSH Hyperproduction Due to INHA Mutation in Primary Ovarian Insufficiency |
|  | BMP Signaling Impairment in Granulosa Cell in POF |
|  | Prostate Cancer |
|  | Cytokines Trigger Otitis Media |
|  | Podocyte Dysfunction in Glomerulonephritis |
|  | Mesangial Cell Dysfunction in Glomerulonephritis |
|  | Interstitial Fibroblasts in Pyelonephritis |
|  | Proteins Involved in Cryptorchidism |
|  | BMP6/ACVR2A Expression Targets |
|  | BMP7/ACVR2 Expression Targets |
|  | Dexamethasone Induced Osteoporosis |
|  | N2 Neutrophils in Tumor-Promoting Inflammation and Tumor Progression |
|  | TGFB Signaling activation by Blocking of Tumor Suppressors |
|  | Genes with Mutations in Cancer-Associated Evading of Growth Suppressors |
|  | Proteins with Altered Expression in Cancer-Associated Evading of Growth Suppressors |
|  | HIF1A in Vasculogenic Mimicry of Cancer |
|  | FBLN5 Potentiates Migration and Proliferation of Endothelial Cells in Cancer |
|  | TGFB Family in Epithelial to Mesenchymal Transition in Cancer |
|  | Skeletal Muscle Wasting in Cancer Cachexia |
|  | INHBB/ACVR2 Expression Target |
|  | INHBA/ACVR2/ACVR1 Expression Targets |
|  | NOTCH -> Expression Targets in Thymus |
|  | TGFBR/BMPR -> SMAD2/3 Signaling |
|  | ActivinR -> SMAD2/3 Signaling |
|  | TGFBR -> MEF/MYOD/MYOG Signaling |
|  | TGFBR1/AP-1 Signaling |
|  | MSTN/ACVR2/ACVR1 Expression Targets |
|  | NODAL/ACVR2B Expression Targets |
|  | TGF-beta Signaling |
|  | TGFB2-TGFBR1 Expression Targets |
|  | TGFB3-TGFBR1 Expression Targets |
|  | TGFB1-TGFBR1 Expression Targets |
|  | TGFB1-TGFBR1/AP-1 Expression Targets |
|  | Elevated Receptors -> Expression Targets in Kidney |
|  | New Pathway |
|  | 1\_Differentiation of psoriatic T cells |
|  | 3\_TGFB signaling provokes endochondral ossification with osteophyte formation |
|  | 3\_The role of interstitial fibroblasts in pyelonephritis |
|  | 3\_Mesangial cell dysfunction in glomerulonephritis |
|  | 2\_Podocyte dysfunction in glomerulonephritis |
|  | 1\_Disruption of luteinizing hormone and follicle-stimulating hormone secretion in PCOS |
|  | 3\_Increased proliferation of pulmonary artery smooth muscle cells (PASMCs) in familial forms of pulmonary hypertension |
|  | 2\_Cardiomyocyte hypertrophy |
|  | 3\_Cardiomyocyte dysfunction unrelated to cell junctions in ARVD |
|  | Differentiation of psoriatic T cells |

---

|  |  |
| --- | --- |
| Group | Genes with Mutations Associated with Colorectal Cancer |

---

|  |  |
| --- | --- |
| MedScan ID | 4087 |

---

|  |  |
| --- | --- |
| LocusLink ID | 4087 |
|  | 17126 |
|  | 29357 |
|  | 319898 |

---

|  |  |
| --- | --- |
| Alias | transcription factor Smad2 |
|  | Mothers against DPP homolog 2 |
|  | MGC34440 |
|  | Mad-related protein 2 |
|  | Smad2 transcription factor |
|  | Smad 2 |
|  | JV18-1 |
|  | Mothers against decapentaplegic, Drosophila, homolog of, 2 |
|  | LOC654050 |
|  | RIKEN cDNA 7120426M23 gene |
|  | mother against DPP homolog 2 |
|  | MADR2 |
|  | 7120426M23Rik |
|  | hMAD II |
|  | Mothers against decapentaplegic, Drosophila, homolog of, II |
|  | MAD homolog 2 (Drosophila) |
|  | MAD (mothers against decapentaplegic, Drosophila) homolog II |
|  | hMAD IIs |
|  | MAD homolog II |
|  | MAD (mothers against decapentaplegic Drosophila) homolog 2 |
|  | sma- and mad-related protein 2 |
|  | JV18 I |
|  | Mothers against DPP homolog II |
|  | mMad2 |
|  | MAD, mothers against decapentaplegic homolog 2 (Drosophila) |
|  | Madh2 |
|  | SMAD, mothers against DPP homolog II |
|  | MAD (mothers against decapentaplegic homolog II |
|  | SMAD, mothers against DPP homolog 2 |
|  | homolog of Drosophila Mothers against decapentaplegic,, 2 |
|  | MGC22139 |
|  | Mothers against decapentaplegic homolog II |
|  | MAD (mothers against decapentaplegic homolog 2 |
|  | Mothers against decapentaplegic homolog 2 |
|  | JV18 |
|  | Mad-related protein II |
|  | hMAD-2 |
|  | MAD homolog 2 |
|  | 7120426M23 |
|  | homolog of Drosophila Mothers against decapentaplegic,, II |
|  | LOC653804 |
|  | MGC156656 |
|  | hSMAD2 |
|  | Smad-2 |

---

|  |  |
| --- | --- |
| GO ID | 0003677 |
|  | 0001228 |
|  | 0003700 |
|  | 0000981 |
|  | 0070411 |
|  | 0070412 |
|  | 0000978 |
|  | 0046332 |
|  | 0033613 |
|  | 0003682 |
|  | 0070410 |
|  | 0097718 |
|  | 0003690 |
|  | 0035326 |
|  | 0046872 |
|  | 0019902 |
|  | 0070878 |
|  | 0046982 |
|  | 0042803 |
|  | 0048156 |
|  | 0008134 |
|  | 0005160 |
|  | 0030618 |
|  | 0034713 |
|  | 0031625 |
|  | 0007183 |
|  | 0060395 |
|  | 0032924 |
|  | 0030325 |
|  | 0009952 |
|  | 0045165 |
|  | 0007182 |
|  | 0048701 |
|  | 0048617 |
|  | 0001706 |
|  | 0007369 |
|  | 0001701 |
|  | 0030073 |
|  | 0035556 |
|  | 0030324 |
|  | 0001707 |
|  | 0008285 |
|  | 0000122 |
|  | 0045892 |
|  | 0030512 |
|  | 0038092 |
|  | 0035265 |
|  | 0031016 |
|  | 0048340 |
|  | 0060039 |
|  | 0030513 |
|  | 0010718 |
|  | 1900224 |
|  | 0045944 |
|  | 0045893 |
|  | 0009791 |
|  | 0031053 |
|  | 0016579 |
|  | 0051098 |
|  | 0017015 |
|  | 0070723 |
|  | 0009749 |
|  | 0062009 |
|  | 0023019 |
|  | 0035019 |
|  | 0007179 |
|  | 0001657 |
|  | 0042060 |
|  | 0007352 |
|  | 0071141 |
|  | 0032444 |
|  | 0005737 |
|  | 0005829 |
|  | 0071144 |
|  | 0000790 |
|  | 0005654 |
|  | 0005634 |
|  | 0032991 |
|  | 0005667 |
|  | 0048589 |
|  | 0009880 |
|  | 0007492 |
|  | 0007507 |
|  | 0010629 |
|  | 0007389 |
|  | 0010628 |
|  | 0006468 |
|  | 0006355 |
|  | 0005622 |
|  | 0005623 |
|  | 0005515 |
|  | 0043234 |
|  | 0060021 |
|  | 0006351 |
|  | 0001077 |
|  | 0010467 |
|  | 0006366 |
|  | 0006367 |

---

|  |  |
| --- | --- |
| KEGG ID | hsa:4087 |
|  | mmu:17126 |
|  | rno:29357 |

---

|  |  |
| --- | --- |
| Organism | Homo sapiens {Organism urn:agi-taxid:9606} |
|  | Mus musculus {Organism urn:agi-taxid:10090} |
|  | Rattus norvegicus {Organism urn:agi-taxid:10116} |
|  | Mus musculus |
|  | Homo sapiens |
|  | Rattus norvegicus |

---

|  |  |
| --- | --- |
| Mouse chromosome position | 18 51.42 cM |
|  | 18 48.0 cM |

---

|  |  |
| --- | --- |
| OMIM ID | 601366 |

---

|  |  |
| --- | --- |
| Rat chromosome position | 18q12.3 |

---

|  |  |
| --- | --- |
| Hugo ID | 6768 |
|  | HGNC:6768 |

---

|  |  |
| --- | --- |
| Human chromosome position | 18q21.1 |

---

|  |  |
| --- | --- |
| Swiss-Prot Accession | Q15796 |
|  | Q53XR6 |
|  | B7Z5N5 |
|  | Q15796.1 |
|  | Q62432 |
|  | Q62432.2 |
|  | O70436 |
|  | A0A0G2K0P2 |
|  | O70436.1 |
|  | Q6GU18 |
|  | Q6VP00 |
|  | Q9D8P6 |
|  | A0JPM1 |

---

|  |  |
| --- | --- |
| PIR ID | S71797 |

---

|  |  |
| --- | --- |
| GenBank ID | NC\_000018 |
|  | NM\_001003652 |
|  | NP\_001003652 |
|  | NM\_005901 |
|  | NP\_005892 |
|  | NM\_001135937 |
|  | NP\_001129409 |
|  | XM\_017025747 |
|  | XP\_016881236 |
|  | XM\_017025746 |
|  | XP\_016881235 |
|  | XM\_011525985 |
|  | XP\_011524287 |
|  | XM\_017025745 |
|  | XP\_016881234 |
|  | XM\_017025750 |
|  | XP\_016881239 |
|  | XM\_006722451 |
|  | XP\_006722514 |
|  | XM\_017025748 |
|  | XP\_016881237 |
|  | XM\_024451173 |
|  | XP\_024306941 |
|  | XM\_011525984 |
|  | XP\_011524286 |
|  | XM\_005258259 |
|  | XP\_005258316 |
|  | XM\_017025749 |
|  | XP\_016881238 |
|  | NG\_029946 |
|  | AC026898 |
|  | AC120349 |
|  | AH006488 |
|  | CH471096 |
|  | EAW62914 |
|  | EAW62915 |
|  | EAW62916 |
|  | EAW62917 |
|  | AA081871 |
|  | AA418737 |
|  | AF027964 |
|  | AAC51918 |
|  | AI087928 |
|  | AI453799 |
|  | AI765747 |
|  | AK126373 |
|  | AK299218 |
|  | BAH12971 |
|  | AK312963 |
|  | BAG35802 |
|  | AY134745 |
|  | AAN08619 |
|  | BC014840 |
|  | AAH14840 |
|  | BC025699 |
|  | AAH25699 |
|  | BI258844 |
|  | BQ018815 |
|  | BQ900993 |
|  | BT007422 |
|  | AAP36090 |
|  | BU167620 |
|  | BU517178 |
|  | BX491944 |
|  | CR992221 |
|  | DB035686 |
|  | DB462454 |
|  | DC371139 |
|  | U59911 |
|  | AAC50789 |
|  | U65019 |
|  | AAB17054 |
|  | U68018 |
|  | AAB17087 |
|  | Q15796 |
|  | NC\_000084 |
|  | XM\_017317842 |
|  | XP\_017173331 |
|  | NM\_010754 |
|  | NP\_034884 |
|  | XM\_006525699 |
|  | XP\_006525762 |
|  | NM\_001252481 |
|  | NP\_001239410 |
|  | NM\_001311070 |
|  | NP\_001297999 |
|  | AC107768 |
|  | AC164009 |
|  | CH466528 |
|  | EDL09484 |
|  | EDL09485 |
|  | AF005743 |
|  | AAB62269 |
|  | AK007817 |
|  | BAB25282 |
|  | AK031111 |
|  | AK078612 |
|  | AK088249 |
|  | BAC40236 |
|  | AV232667 |
|  | AY334552 |
|  | AAR00933 |
|  | BC021342 |
|  | AAH21342 |
|  | BC058713 |
|  | BC089184 |
|  | AAH89184 |
|  | BM248478 |
|  | BU058005 |
|  | CN456711 |
|  | U60530 |
|  | AAB03612 |
|  | Q62432 |
|  | NC\_005117 |
|  | XM\_006254945 |
|  | XP\_006255007 |
|  | NM\_001277450 |
|  | NP\_001264379 |
|  | NM\_019191 |
|  | NP\_062064 |
|  | XM\_006254946 |
|  | XP\_006255008 |
|  | AC\_000086 |
|  | AABR07032580 |
|  | AABR07032581 |
|  | AAHX01096051 |
|  | AAHX01096052 |
|  | CH474069 |
|  | EDL84731 |
|  | EDL84732 |
|  | EDL84733 |
|  | EDL84734 |
|  | AB010147 |
|  | BAA81909 |
|  | AB017912 |
|  | BAA33453 |
|  | AF056001 |
|  | AAC12780 |
|  | BC127497 |
|  | AAI27498 |
|  | FQ230791 |
|  | O70436 |
|  | DQ891501 |
|  | U78733 |
|  | U78732 |
|  | DQ894693 |
|  | XM\_011525986 |
|  | U78729 |
|  | XM\_011525983 |
|  | U78726 |
|  | XM\_006525700 |
|  | U78728 |
|  | U78727 |
|  | AAC39657 |
|  | U78731 |
|  | U78730 |
|  | DQ893746 |
|  | AABR06096004 |
|  | AABR06096003 |
|  | ABM84672 |
|  | XP\_011245156 |
|  | AC\_000040 |
|  | ABM82427 |
|  | AC\_000150 |
|  | AAHY01140355 |
|  | XM\_005258260 |
|  | AAHY01140350 |
|  | AMYH02034675 |
|  | AAHY01140352 |
|  | AMYH02034676 |
|  | AAHY01140351 |
|  | AAHY01140354 |
|  | AAHY01140353 |
|  | ABM84458 |
|  | XP\_006525763 |
|  | AMYH02034674 |
|  | NC\_018929 |
|  | XM\_011246854 |
|  | XP\_005258317 |
|  | ABM85619 |
|  | XP\_011524285 |
|  | XP\_011524288 |
|  | ABBA01018627 |
|  | ABBA01018626 |
|  | DQ893532 |

---

|  |  |
| --- | --- |
| Swiss-Prot ID | SMAD2\_HUMAN |
|  | SMAD2\_MOUSE |
|  | SMAD2\_RAT |

---

|  |  |
| --- | --- |
| Cell Localization | Cytoplasm |
|  | Nucleus |

---

|  |  |
| --- | --- |
| Ensembl ID | ENSG00000175387 |
|  | ENSP00000384449.1 |
|  | ENST00000402690.6 |
|  | ENSP00000349282.4 |
|  | ENST00000356825.8 |
|  | ENSP00000262160.6 |
|  | ENST00000262160.11 |
|  | ENSP00000466254.1 |
|  | ENST00000587269.5 |
|  | ENSMUSG00000024563 |
|  | ENSMUSP00000025453.8 |
|  | ENSMUST00000025453.14 |
|  | ENSMUSP00000109563.2 |
|  | ENSMUST00000113930.2 |
|  | ENSMUSP00000130115.1 |
|  | ENSMUST00000168423.8 |
|  | ENSMUSP00000089439.6 |
|  | ENSMUST00000091831.12 |
|  | ENSRNOG00000018140 |
|  | ENSRNOP00000048329.3 |
|  | ENSRNOT00000046847.4 |
|  | ENSRNOP00000075341.1 |
|  | ENSRNOT00000092173.1 |
|  | ENSRNOP00000071524.1 |
|  | ENSRNOT00000089468.1 |
|  | ENSMUST00000091831 |
|  | ENSRNOP00000048329 |
|  | ENSRNOP00000071524 |
|  | ENST00000586040 |
|  | ENSMUST00000168423 |
|  | ENSP00000349282 |
|  | ENSP00000262160 |
|  | ENSMUST00000025453 |
|  | ENSP00000384449 |
|  | ENST00000262160 |
|  | ENSRNOT00000046847 |
|  | ENSRNOP00000075341 |
|  | ENSRNOT00000089468 |
|  | ENSMUSP00000025453 |
|  | ENSP00000466193 |
|  | ENSMUST00000168423.7 |
|  | ENSMUSP00000130115 |
|  | ENST00000356825 |
|  | ENST00000402690 |
|  | ENST00000262160.10 |
|  | ENSRNOT00000092173 |
|  | ENSMUSP00000089439 |

---

|  |  |
| --- | --- |
| MGI ID | MGI:108051 |
|  | 108051 |

---

|  |  |
| --- | --- |
| RGD ID | 3031 |

---

|  |  |
| --- | --- |
| Unigene ID | Hs.705764 |
|  | Mm.152699 |
|  | Hs.741342 |
|  | Rn.2755 |
|  | Hs.12253 |
|  | Mm.490934 |

---

|  |  |
| --- | --- |
| Homologene ID | 21197 |

---

|  |  |
| --- | --- |
| Shape | O-vertex |

---

|  |  |
| --- | --- |
| IPI ID | IPI00019548 |
|  | IPI00458087 |
|  | IPI00220364 |
|  | IPI00198544 |
|  | IPI00556692 |

---
